# Combining epigenetic and clinicopathological variables improves specificity in prognostic prediction in clear cell renal cell carcinoma

**DOI:** 10.1186/s12967-020-02608-1

**Published:** 2020-11-13

**Authors:** Emma Andersson-Evelönn, Linda Vidman, David Källberg, Mattias Landfors, Xijia Liu, Börje Ljungberg, Magnus Hultdin, Patrik Rydén, Sofie Degerman

**Affiliations:** 1 Department of Medical Biosciences, Pathology, Umeå University, 901 87 Umeå, Sweden; 2 Department of Mathematics and Mathematical Statistics, Umeå University, 901 87 Umeå, Sweden; 3 Department of Statistics, USBE, Umeå University, Umeå, Sweden; 4 Department of Surgical and Perioperative Sciences, Urology and Andrology, Umeå University, Umeå, Sweden; 5 Department of Clinical Microbiology, Umeå University, Umeå, Sweden

**Keywords:** Clear cell renal cell carcinoma, Classification, DNA methylation, Prognosis, Directed cluster analysis

## Abstract

**Background:**

Metastasized clear cell renal cell carcinoma (ccRCC) is associated with a poor prognosis. Almost one-third of patients with non-metastatic tumors at diagnosis will later progress with metastatic disease. These patients need to be identified already at diagnosis, to undertake closer follow up and/or adjuvant treatment. Today, clinicopathological variables are used to risk classify patients, but molecular biomarkers are needed to improve risk classification to identify the high-risk patients which will benefit most from modern adjuvant therapies. Interestingly, DNA methylation profiling has emerged as a promising prognostic biomarker in ccRCC. This study aimed to derive a model for prediction of tumor progression after nephrectomy in non-metastatic ccRCC by combining DNA methylation profiling with clinicopathological variables.

**Methods:**

A novel cluster analysis approach (Directed Cluster Analysis) was used to identify molecular biomarkers from genome-wide methylation array data. These novel DNA methylation biomarkers, together with previously identified CpG-site biomarkers and clinicopathological variables, were used to derive predictive classifiers for tumor progression.

**Results:**

The “triple classifier” which included both novel and previously identified DNA methylation biomarkers together with clinicopathological variables predicted tumor progression more accurately than the currently used Mayo scoring system, by increasing the specificity from 50% in Mayo to 64% in our triple classifier at 85% fixed sensitivity. The cumulative incidence of progress (_p_CIP_5yr_) was 7.5% in low-risk vs 44.7% in high-risk in M0 patients classified by the triple classifier at diagnosis.

**Conclusions:**

The triple classifier panel that combines clinicopathological variables with genome-wide methylation data has the potential to improve specificity in prognosis prediction for patients with non-metastatic ccRCC.

## Background

More than a hundred thousand individuals in Europe are yearly diagnosed with kidney cancer [1, 2]. Renal cell carcinoma (RCC) is the most common type of kidney cancer and clear cell RCC (ccRCC) constitutes 75% of RCC cases [3]. Due to more extensive use of computed imaging, ultrasound, and magnetic resonance in diagnostics most patients with RCC are diagnosed incidentally before patients experience any classic symptoms (i.e. flank pain, haematuria, or a palpable abdominal mass) [4] and the frequency of metastatic disease at diagnosis has therefore decreased from 30 to 18% over a 5-year period (2005 to 2009) [5]. Patients with non-metastatic disease at diagnosis have a 5-year survival of 75–85%, in contrast to only 10% in patients with metastatic disease. Among patients with non-metastatic disease at diagnosis, approximately 20–30% will later develop metastasis after nephrectomy, i.e. progression of the disease [5].

Non-metastatic patients with a high risk of tumor progression might benefit from an intensified follow-up to improve early diagnosis of tumor recurrence, and in the long run, be candidates for adjuvant treatment. Therefore, it is essential to identify those patients already at diagnosis by reliable biomarkers with high sensitivity and specificity.

Currently, the Mayo scoring system (Mayo) is used to predict outcome and treatment stratification in ccRCC [6]. Mayo combines T-stage, N-stage, tumor size, Fuhrman grade, and the presence of necrosis to create a score dividing patients into three groups: low, intermediate, and high-risk for tumor progression. Depending on the risk group, the frequency and the total follow-up time differs, ranging from 5 to 10 years [7].

Altered DNA methylation has been identified as a prognostic marker in several malignancies including ccRCC, and has been suggested as a potential target for therapy [8, 9]. We have recently shown that increased promoter-associated DNA methylation is correlated to poorer outcomes in ccRCC [10, 11]. Studies by others have identified selected CpGs with potential relevance for ccRCC prognosis [12–17]. Arai et al. and Tian et al. identified CpG island methylator phenotype (CIMP) panels, that predicted cancer-free survival and overall survival [13, 18]. In 2015, Wei et al. presented a risk score based on five CpGs that predicted overall survival in three different ccRCC cohorts [14]. Joosten et al. [12] performed a systematic review summarizing prognostic DNA methylation biomarkers in ccRCC, and identified nine genes with strong evidence as prognostic biomarkers in ccRCC. However, few of these panels which are based on a limited number of CpGs, did show reproducible prognostic relevance in predicting risk for progress in non-metastatic patients [11]. Larger genome-wide panels of CpG sites might be needed for improved risk classification [12].

An interesting approach suggested by Thompson and Marsit [19] combines several molecular and clinical biomarkers to create a prognostic risk score for ccRCC patients. Even though the benefits of integrative analysis using different omics measurements and clinical data have started to become clear, few current risk classification models combine clinicopathological variables with molecular biomarkers.

The current project aimed to derive a classifier to estimate the risk of tumor progression after nephrectomy in patients with local ccRCC at diagnosis, by combining genome-wide methylation profiles and established clinicopathological variables. Three sets of biomarkers were considered in the prognostic modeling: clinicopathological variables, previously identified CpGs (PI-CpGs), and a set of methylation biomarkers obtained using a novel approach called Directed Cluster Analysis (DCA). These sets were used together with logistic regression to build classifiers predicting patients as either high risk for progress (HRP) or low risk for progress (LRP).

## Methods

### ccRCC patient samples

This study included 115 patients with ccRCC, diagnosed between 2001 and 2009 at the University Hospital in Umeå, Sweden. All patients were primarily treated with either radical or partial nephrectomy and followed up in accordance with the Swedish health care program for kidney cancer (median follow-up time 98 months ranging from 1–193 months) [7]. No patient included in this study received neoadjuvant and no non-metastatic patients received adjuvant treatment. Tissue samples were obtained after nephrectomy, snap-frozen in liquid nitrogen, and stored at − 80 °C until analysis. DNA was extracted from tissue samples as described previously [20].

The tumor disease was classified using the tumor-node-metastasis (TNM) classifications system [21]. Eighty-seven patients were non-metastatic (M0 i.e. TNM I-III) and 28 patients had metastatic disease (M1 i.e. TNM IV) at diagnosis. The nuclear grading was performed according to Fuhrman et al. [22] and the largest tumor diameter was measured at the CT scans. Blood samples were taken within a week before nephrectomy and analyzed for hemoglobin levels, thrombocyte particle count, calcium concentration, albumin levels, gamma-glutamyltransferase, alkaline phosphatase, and creatinine levels. The above variables were included in the analysis and are henceforth referred to as the clinical variables. Here, missing values were imputed using the mean value.

All tumors were classified according to the Mayo scoring system, which calculates a risk score based on T-stage (T1a, T1b, T2, and T3-T4), tumor size (smaller/larger than 10 cm), N-stage (NX/N0, N1), Fuhrman grade (Grade 1–2, Grade 3, Grade 4) and tumor necrosis (absent/present) (Additional file 1: Table S1) [6, 7].

Clinical follow-up data were extracted in August 2017. All patients have given informed and signed consent and the study was approved by the regional ethical review board in Umeå (Dnr 2011–156-31 M, 20110523).

### High-dimensional DNA methylation arrays

The DNA methylation analysis has previously been described by Evelönn et al. [11]. In short; DNA was extracted from tissue samples, from 115 tumors and 12 tumor-free (TF) tissue samples, and bisulfite converted using the EZ DNA Methylation Kit (Zymo Research, Irvine, USA). Methylation conversion was verified by MethyLight analysis [23] and samples were assessed for DNA methylation using HumanMethylation450K BeadChip arrays (Illumina, San Diego, Ca, USA). The arrays were scanned with the HiScan array reader (Illumina). The quality of each array was evaluated with the built-in controls. The technical reproducibility of methylation array analysis was monitored by replicated samples on each array, and the R2-values ranged from 0.995 to 0.997.

Pre-processing was performed as previously described by Degerman et al. [24]. Briefly, the fluorescence intensities were extracted using the Methylation Module (1.9.0) in the Genome Studio software (V2011.1). Pre-processing and downstream analysis was done using R (v2.15.0 and v3.4.3). Data were normalized using the BMIQ method [25] to compensate for the two different bead types used in the array. Filtering of data included the exclusion of CpG probes; located at the X and Y chromosomes, that align to multiple loci in the genome [26], located less than 3 bp from a known single nucleotide polymorphism [26], without representation on the Illumina EPIC methylation arrays or had a detection p-value larger than 0.05 or a signal from less than 3 nbeads in any sample. The analysis was focused on CpGs located within the promoter region close to the transcription start site (TSS) (i.e. the regions denoted TSS1500, TSS200, 5′UTR, and exon 1). CpGs outside these regions and CpGs located in methylation quantitative trait loci (mQTLs) [27] were also excluded from the analysis. The methylation level (β-value) of each CpG site ranged from 0 (no methylation) to 1 (complete methylation). Differently methylated CpG sites (DM-CpGs) were identified by comparing the sample’s β-value with the average β-value taken over the twelve TF-samples, and an absolute Δβ_tumor-TF-tissue_-value ≥ 0.2 was considered as a DM-CpG.

### Selection of CpGs previously associated with ccRCC prognosis

CpG sites previously associated with ccRCC, and present on HumMeth450K arrays, where identified in five original publications and one review and included in the analysis [12–17]. The systematic review by Joosten et al. 2017, identified nine genes with strong evidence as prognostic DNA methylation-based biomarkers in ccRCC [12]. Ricketts et al. [15] listed genes known to be hypermethylated in ccRCC in the publically available Tumor Cancer Genome Atlas Kidney Renal Cell Carcinoma (TCGA-KIRC) project. Forty-five genes met the criterion to be hypermethylated in relation to paired tumor-free samples, and the CpG presented as most hypermethylated for each gene was included in our analysis. Van Vlodrop et al. [16] identified a four-gene promoter methylation marker panel that was associated with cancer-specific survival and validated in the TCGA-KIRC cohort. Wei et al. [14] analyzed paired tumor and tumor-free tissue samples from 46 individuals on the Infinium HumMeth450K arrays and built a model including five CpGs estimating risk for death. Arai et al. [13] created a 16 CpG site CIMP classification panel using the Infinium 27 K arrays that was correlated to more aggressive tumors. Fourteen out of these CpGs were represented on the Infinium HumMeth450K arrays and included in our analysis. Wang et al. [17] identified a CpG site in the DAB2IP gene, both by Illumina 450 K arrays and pyrosequencing that was correlated to poorer overall survival if hypermethylated.

The CpG sites identified in these publications include 87 CpGs of which 78 were unique, nine CpGs were presented in more than one publication (Additional file 2: Table S2). All CpG biomarkers included in our analysis had to fulfill the filtering steps described above except the requirement for being located within a gene promoter. After going through the filtration steps, 64 CpG sites previously associated with ccRCC prognosis remained and were included in the analysis (Additional file 3: Table S3).

### Directed cluster analysis

**Fig. 1:**
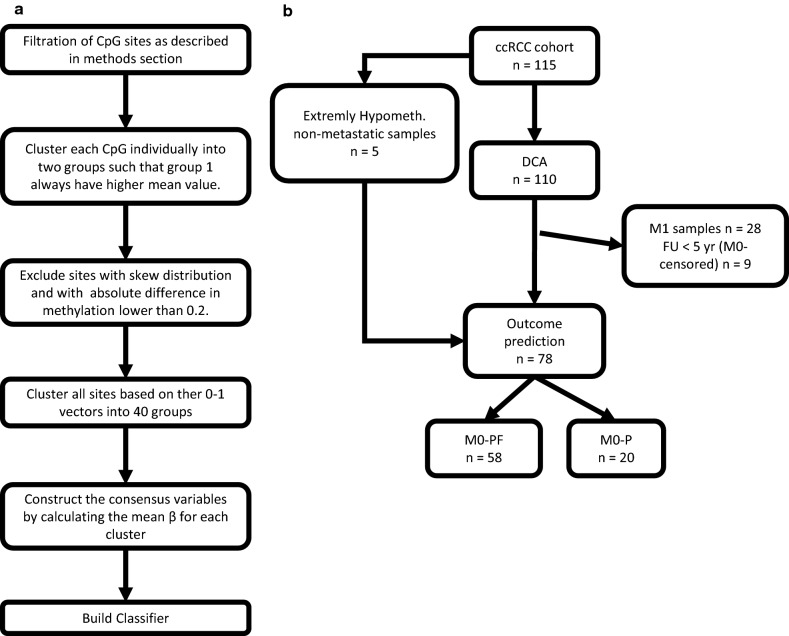
Analysis workflow. **a** DCA consensus cluster workflow. Showing the steps for the creation of consensus variables using Directed Cluster Analysis (DCA). **b** Patient inclusion in analysis steps

CpG clusters of potential prognostic relevance were identified using the novel clustering method Directed Cluster Analysis (DCA) described below. For each CpG site, the samples were grouped using 2-means clustering. Samples belonging to the group with the highest mean β-value were labeled 1 while the other samples were labeled 0. Hence, for each CpG site, a binary profile vector was constructed. CpGs with less than 10% of the samples in the smallest group were removed from further analysis. The difference between the mean β-values in the two groups was calculated for each CpG and sites with an absolute difference lower than 0.2 were excluded from downstream analysis. The remaining CpGs were clustered using k-means with k = 40. Note that CpGs with similar binary partition of the samples are more likely to end up in the same cluster. For each cluster and sample, a robust consensus variable was obtained by calculating the mean β-value of all sites included in the cluster. The consensus variables were treated as biomarkers. The DCA method workflow is summarized in Fig. 1a, b.

### Classification

The classification was used to estimate the risk of metastasis within 5 years among patients with non-metastatic disease at diagnosis. The inclusion criterion was at least a 5-year follow-up, which was met by 78 non-metastatic patients. Fifty-eight patients remained non-metastatic (M0-PF), while 20 patients progressed (M0-P) during the 5-year follow-up. Three sets of biomarkers were considered: the clinicopathological variables (clinical), the previously identified ccRCC associated methylation sites (PI-CpGs), and the consensus variables identified by directed cluster analysis (DCA). The sets were used individually and combined in six sets: clinical, PI-CpGs, DCA, clinical + PI-CpGs, clinical + DCA, and clinical + PI-CpGs + DCA. For each set, a classifier was built on the first five Principal Components (PC) using logistic regression. The PCs’ were obtained using standardized variables, except for the categorical and ordinal variables that were mean-centered. The classifiers were evaluated by the specificity observed at a fixed 85% sensitivity.

### Statistical analysis

Differences between patient groups were analyzed using the Mann–Whitney U test for continuous variables and the Chi-square test for categorical variables. The calculations were performed in R v3.4.3. Agreement between the Mayo (MAYO_high/intermediate_ and MAYO_low_ risk) and the triple classifier (high risk and low risk) was measured by the Cohen´s kappa score in R-package psych.

Receiver operating characteristic (ROC) analysis and the area under the curve (AUC) calculations were done using R-package pROC, Mayo scores, and the posterior probabilities of the six classifiers. The ROC curves were compared using the methodology described by DeLong et al. [28].

The cumulative incidence of progress within 5 years from diagnosis (CIP_5yr_) was estimated by Kaplan–Meier survival tables. Differences in survival distributions between subgroups of samples were calculated using the log-rank test. Survival analysis was performed using the Statistical Package for the Social Sciences (SPSS Inc., Chicago, IL) software version 24.

## Results

### Descriptive analysis of the ccRCC cohort

A total of 115 patients were included in this study, 57% were men, and the median age was 65 (± 11.6) years at diagnosis. The patients were divided into three groups; non-metastatic progression free (M0-PF, n = 58); non-metastatic with progression within 5 years (M0-P; n = 20), and metastasized at diagnosis (M1; n = 28). Nine non-metastatic patients (M0-censored) were excluded from the risk classification models as 5 years follow-up was not reached e.g. due to the death of other diseases (Fig. 1b). No significant differences in age or gender were observed between the subgroups (Table 1).

**Table 1:** Clinicopathological variables and their relation to ccRCC progression

Variable	M0-PF n = 58	M0-P n = 20	M1 n = 28	M0-PF vs M0-P p-value	M0-PF vs M1 p-value	M0-P vs M1 p-value
Gender						
Male	32	10	21	0.888	0.125	0.139
Female	26	10	7			
Morphological grade^a^						
G1	12	1	0	0.002	< 0.001	0.394
G2	30	6	7			
G3	15	8	8			
G4	1	5	12			
TNM						
I	39	5	0	< 0.001	< 0.001	< 0.001
II	10	2	0			
III	9	13	0			
IV	0	0	28			
Mayo						
Low	29	3		0.006	–	–
Intermediate/high	29	17				
Age (years)	65.6 (11.6)	64.2 (11.9)	63.0 (11.0)	0.590	0.345	0.706
Albumin (g/L)	40.6 (4.1)	38.6 (7.2)	36.7 (4.5)	0.299	< 0.001	0.121
Alkaline phosphatase (µkat/L)^b^	1.9 (2.0)	2.2 (1.5)	5.1 (6.6)	0.745	< 0.001	0.026
Calcium (mmol/L)^c^	2.35 (0.14)	2.32 (0.13)	2.46 (0.26)	0.358	0.212	0.129
Creatinine (µmol/L)	79.6 (16.3)	86.0 (32.4)	93.3 (27.6)	0.837	0.015	0.300
Gamma glutamyltanseferase (µkat/L)^d^	0.76 (1.17)	0.83 (1.21)	2.23 (3.06)	0.401	< 0.001	0.002
Hemoglobin (g/L)	137.6 (17.3)	121.1 (22.0)	116.9 (19.1)	0.002	< 0.001	0.331
Thromobocyte particle count (10^9^/L)	251.7 (77.7)	316.5 (175.4)	365.3 (136.8)	0.112	< 0.001	0.090
Tumor diameter (mm)	56.5 (31.2)	95.0 (44.8)	108.3 (35.7)	< *0.001*	< *0.001*	*0.262*

Mean values and standard deviation (SD) are reported for each continuous variable. Chi-square tests were used for testing independence between categorical variables and Mann–Whitney U tests were used for comparisons between continuous variables. M0 and M1 denote non-metastatic and metastatic patients at diagnosis respectively, while PF and P denote progress free patients and patients with progress within 5 years respectively

^a^One missing value in group M1

^b^Three missing values. One value is missing in each of M0-PF, M0-P and M1

^c^One missing value in M0-PF

^d^Four missing values. Three in M0-PF and one in M1

### Individual analysis of clinical and methylation variables.

Twelve clinical variables were compared in the groups M0-PF and M0-P. The tumor diameter was significantly larger in the M0-P group than in the M0-PF group at diagnosis (p < 0.001), 95.0 mm and 56.5 mm, respectively. Moreover, the distributions of morphological grade (p = 0.002) and TNM stage (p < 0.001) differed in M0-PF and M0-P patients, with a higher grade/TNM stage in the M0-P patients. The hemoglobin value was significantly higher (p = 0.002) in M0-PF (mean 137.6 g/l) than in M0-P (mean 121.1 g/l) patients at diagnosis. For the remaining clinical variables, no significant differences were observed (Table 1).

Next, differences in methylation between M0-P and M0-PF were investigated. For the 64 previously identified sites (PI-CpGs), 15 (23.4%) sites were significantly more methylated in the M0-P group compared to the M0-PF group (Additional file 3: Table S3).

Before the DCA analysis, an overview of all the samples’ methylation profiles was performed. Five non-metastatic samples showed an extreme number of hypomethylated (Δβ ≤ − 0,2) CpGs and were excluded from the DCA clustering process to avoid heavily unbalanced distributions in the first step in the DCA procedure. However, these samples were included in the downstream analysis after the consensus variables were constructed (Fig. 1b).

Applying the DCA method yielded 40 DNA methylation cluster variables (Additional file 4: Table S4). Six (15%) of these variables showed a significantly higher methylation level in the M0-P group compared to the M0-PF group (Additional file 5: Table S5).

### Classification of non-metastatic ccRCC patients

The objective was to build classification models that can be used to predict tumor progression among non-metastatic ccRCC patients at diagnosis, i.e. either M0-PF or M0-P. Six classification models were constructed and evaluated against the Mayo classification. Mayo classified 32 patients as low-risk, 36 patients as intermediate-risk, and 10 patients as high-risk. To calculate the specificity and sensitivity of Mayo, we pooled the Mayo intermediate and Mayo high groups motivated by identical clinical follow-up scheme for these patients [7]. The pooled group was denoted Mayo intermediate/high. Mayo showed 85% sensitivity and 50% specificity in our cohort.

The six classifiers were derived using logistic regression and the first five principal components taken from one or several of the considered data sets i.e. clinical, PI-CpGs, DCA, clinical + PI-CpGs, clinical + DCA, and clinical + PI-CpGs + DCA. Each classifier predicted the patients as either low risk for progress (LRP) or high risk for progress (HRP) (Additional file 6: Table S6). To enable comparison with Mayo, the classifiers were evaluated by considering the specificity when the sensitivity was fixed at 85% by controlling the cutoff of the predicted posterior probabilities. The expected specificity observed by chance for a non-informative random data was tested through simulation and was estimated to 11%.

The performances of the classifiers using the clinical, PI-CpGs, and DCA data alone were compared with Mayo. The clinical, PI-CpGs, and DCA classifiers had 43%, 59% and 43% specificity respectively (Table 2). The deviated predictions suggest that the data sets contain complementary information and that better predictive models can be obtained by combining several data sets.

**Table 2:** Performance of the considered classifiers and the Mayo Scoring system

	Classifier	Sensitivity (%)	Specificity (%)
A	Mayo scoring system (Mayo)	85	50
B	Clinicopathological variables (Clinical)	85	43
C	Identified prognostic biomarker CpGs (PI-CpGs)	85	59
D	Consensus variables (DCA)	85	43
E	Clinical + PI-CpGs	85	55
F	Clinical + DCA	85	53
G	Clinical + PI-CpGs + DCA (the triple classifier)	85	64

Accordingly, the triple classifier using data from all three data sets, i.e. 12 clinical variables, 64 PI-CpGs, and 40 DCA variables had the highest specificity (64%) (Table 2). This triple classifier correctly identified 37 of 58 patients without progression and 17 of 20 patients with tumor progression. Classification of new ccRCC patients using the triple classifier can be performed using the R script found at: https://github.com/LindaVi/ccRCC-classification. To impute missing values, mean values based on our cohort for all variables included in the model, are supplied along with the script.

ROC-curves were made for all classifiers and no significant differences (p > 0.05 for all pairwise comparisons) were shown between the overall AUC-values (Additional file 8: Figure S1).

### Similarities between models

A sensitivity of 85% entails that each of the models correctly classified 17 of the 20 patients with tumor progression within 5 years. Sixty-five percent of the M0-P patients and 21% of the M0-PF patients were correctly classified by all seven models, including Mayo. Moreover, 47% of the patients (independent of true outcome) were classified the same by all classifiers (Additional file 6: Table S6).

**Fig. 2:**
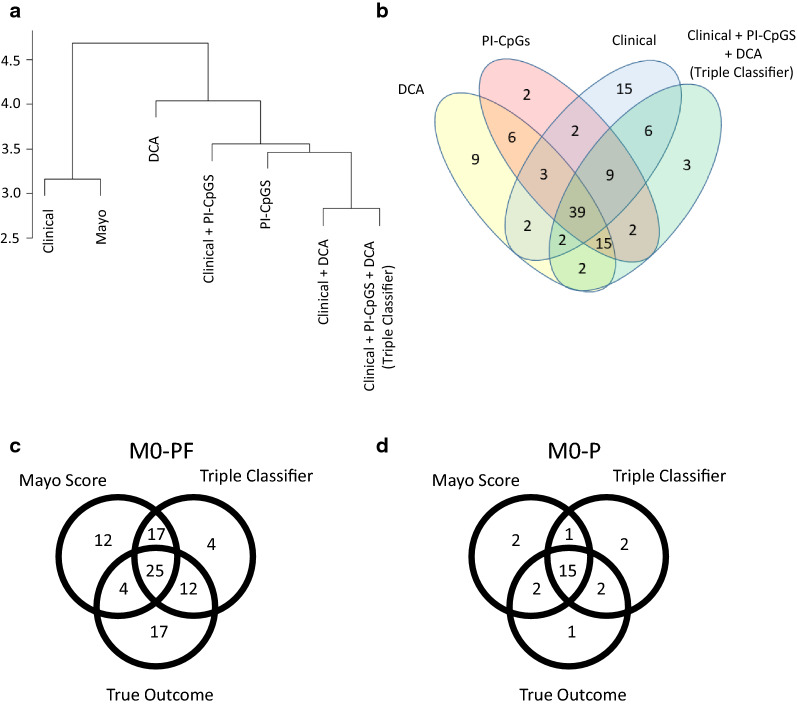
Classification similarities. **a** Clustering of classification results using hierarchical clustering with Euclidean distance and average linkage. **b** The number of samples that were classified identically by the classifiers using Directed Cluster Analysis biomarkers (DCA), previously identified biomarkers (PI-CpGs), clinical variables (clinical), and the triple classifier (clinical + PI-CpGs + DCA)**. c, d** The number of samples that were classified identically by the triple classifier and the Mayo Scoring System for the true outcome in **c** non-metastatic ccRCC at diagnosis and progress-free after 5 years (M0-PF) patients and **d** non-metastatic ccRCC at diagnosis with progression within 5 years (M0-P) patients

As expected, methods relying on only clinical data (clinical and Mayo) gave similar output (Fig. 2a and Additional file 6: Table S6), where 87% of the patients were classified identically by the methods. The DCA and PI-CpGs classifiers that rely on only methylation data, showed identical classification output in 81% of the patients (Fig. 2a, b). The similarities in the classification of the triple classifier compared to every single subset; clinical, PI-CpGs and DCA, showed an overlap in 72%, 83%, and 74% respectively (Fig. 2b).

The triple classifier showed a moderate agreement with the Mayo classification, with Cohen´s kappa = 0.49 and with 74% of the samples classified identically (Fig. 2c, d and Additional file 7: Table S7).

### Survival analysis

**Fig. 3:**
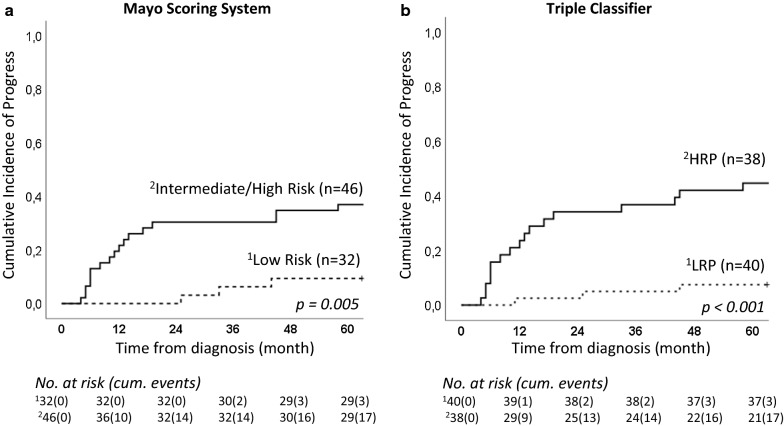
Cumulative incidence of progress (pCIP_5y_). Seventy-eight non-metastatic tumors were classified using **a** the Mayo Scoring System (Mayo) and **b** the triple classifier (clinical + PI-CpGs + DCA) at diagnosis. The pCIP_5y_ were compared in the risk groups. Log-rank *p-*values are presented

Cumulative incidence of progress (CIP) analysis was used to investigate the prognostic relevance for the triple classifier and Mayo in 78 non-metastatic ccRCC patients. Both classifiers divided the patients into two groups: low risk for progress and high risk for progress. The triple classifier was better than Mayo to prognosticate progress (pCIP_5yr_ 9.4% low risk_Mayo_ vs 7.5% LRP_triple classifier_) (pCIP_5yr_ 37% intermediate/high risk_Mayo_ vs 44.7% HRP_triple classifier_) (Fig. 3).

## Discussion

DNA methylation alterations are early events in tumor progression and has appeared as a molecular biomarker candidate for improved risk classification [11–17]. Our study shows that the prediction of tumor progression within 5 years after nephrectomy in non-metastatic ccRCC patients at diagnosis was improved by using bioinformatics modeling to combine clinicopathological variables with methylation signatures.

One-third of non-metastatic ccRCC patients at diagnosis are at risk for later tumor progress [5]. It is of importance to identify patients with a high-risk for tumor progression, since they may benefit from a more intensive follow-up and adjuvant therapy. As reviewed by Bai et al. [29] several adjuvant studies using tyrosine kinase inhibitors (TKI) have been performed in patients with locally advanced non-metastatic RCC. The conclusion drawn from this systematic review is that there might be ccRCC patients that benefit from adjuvant treatment, as described in the S-TRAC study showing longer disease-free time but not for overall survival, while other TKI studies showed no survival benefit [29, 30] Deepened knowledge of ccRCC biology is important to identify new therapeutic strategies for high-risk patients. New ongoing phase 3 adjuvant immunotherapy trials might theoretically be more successful since these therapies unblock the hampering of the patient’s immune cells by the tumors [31].

Risk classification strategies which include both clinical and molecular biomarkers have the potential to improve sensitivity and specificity and more effectively identify the patients at high risk for progress which may benefit from modern adjuvant therapies, despite the common side-effects.

Our population-based ccRCC cohort represents well-characterized patients with long follow-up time. We performed genome-wide methylation analysis on the diagnostic tumor samples and included both M0 and M1 samples in the DCA modelling.

Among the 78 non-metastatic ccRCC at diagnosis, the currently used Mayo showed a sensitivity of 85% and a specificity of 50% in predicting tumor progression. We tested six different classifiers including clinical and/or methylation derived variables at a fixed desirably high sensitivity of 85% to allow comparison with Mayo, and also motivated by the clinical need for high sensitivity in risk classification. The specificity at the fixed sensitivity of 85% was considerably higher for the triple classifier than for Mayo (64% vs. 50%), although the overall ROC curve analysis could not significantly discriminate between the classifiers’ AUC. Moreover, the triple classifier showed a more accurate prediction of progress than Mayo in the cumulative incidence of progress analysis.

Altogether, the addition of methylation biomarkers to the currently used clinical variables improved the prediction of progress among non-metastatic patients at diagnosis. Thompson and Marsit [19] also combined clinical variables and molecular biomarkers, including both methylation and gene-expression profiles. Their study supports our conclusion that methylation analysis has the potential to improve risk classification in ccRCC.

Biomarkers derived from DNA methylation analysis have several advantages, e.g. DNA methylation alterations are stable, do not require adapted sample handling, and DNA is routinely extracted in the clinic. DNA methylation classifiers are in clinical use as a prognostic marker in other malignancies. Jaunmuktane et al. used the classifier presented by Capper et al. in combination with established methods (i.e. histological assessment and conventional molecular testing) to diagnose CNS tumors. Methylation-based classification contributed to a more accurate and clinically relevant diagnosis, most pronounced in unusual, non-specific, or non-representative histological cases [32, 33]. Bioinformatics pipelines for methylation array data analysis in CNS tumors are being introduced at clinical diagnostic laboratories [32, 34]. Although methylation analyses have shown prognostic value in ccRCC [12] they are still not used in clinical diagnosis.

An alternative classifier using gene expression data, ClearCode34, is of prognostic relevance in several ccRCC cohorts as reviewed by Ghatalia and Rathmell [35]. Expression levels of 34 genes were analyzed and tumors were classified as either low-risk ccA or high-risk ccB. Notably, the same tumor could be categorized as both ccA and ccB due to tumor heterogeneity in expression levels. We have previously shown that DNA methylation patterns in ccRCCs are heterogeneous between different patients, but within individual tumors, the methylation pattern is homogenous [11]. This was further supported by the study of Wei et al. who classified multiple samples within each tumor and showed that 90% of tumors were correctly classified by DNA methylation-based classification [14], suggesting DNA methylation as an attractive and stable biomarker.

The proposed DCA-method is a novel unsupervised approach for identifying potential biomarker panels. Hence, the method does not use class information, in our case information on progress, to derive the biomarkers. An advantage with an unsupervised approach is that it can capture biological signatures also when the classes of interest are heterogeneous.

The DCA variables are obtained by taking the average beta-value over a large number of CpGs, which makes them less sensitive to noise compared to single CpG biomarkers. Future studies on independent ccRCC cohorts are of importance for confirming the prognostic relevance of our defined risk classification panel. We encourage researchers to independently evaluate the proposed triple classifier, utilizing standard clinical data and methylation data from either the Infinium Human Methylation 450 k BeadChip or Infinium MethylationEPIC arrays.

We believe that our study shows the potential in combining clinicopathological variables with methylation signatures to improve current risk classification of non-metastatic ccRCC.

### Conclusions

A combination of clinicopathological and epigenetic variables, i.e. DNA methylation, have benefits in predicting survival of patients with ccRCC, compared to the currently used Mayo classifier. The triple classifier has the potential to enhance specificity in identifying ccRCC patients with a high risk of tumor progression and improve treatment stratification.

## Supplementary information

**Additional file 1: Table S1.** The Mayo scoring algorithm (6), used in the clinic to estimate the risk of tumor progression.

**Additional file 2: Table S2.** CpGs with potential relevance for ccRCC prognosis identified in the literature (PI-CpGs).

**Additional file 3: Table S3.** CpGs with potential relevance for ccRCC prognosis identified in the literature (PI-CpGs). Mean beta values are presented for M0-PF and M0-P patients in our cohort, as well as p-values derived using the Mann-Whitney U test.

**Additional file 4: Table S4.**CpGs included in the 40 consensus variables identified by DCA.

**Additional file 5: Table S5.** Mean beta values for the 40 consensus variables identified by DCA for M0-P and M0-PF patients together with p-value comparing average methylation for each cluster using the Mann-Whitney U test.

**Additional file 6: Table S6.** Prediction results for the considered classifiers and the Mayo scoring system for the 78 non-metastatic patients at diagnosis. Each classifier predicted the patients as either low risk for progress (LRP=0) or high risk for progress (HRP=1).

**Additional file 7: Table S7.** Classification results for the Mayo scoring classifier (Low Risk or Intermediate/High Risk) and the triple classifier (LRP or HRP) in relation to the true outcome (M0-PF or M0-P).

**Additional file 8: Figure S1.** Receiver operating characteristic (ROC) analysis and the area under the curve (AUC) analysis based on Mayo scores, and the posterior probabilities of the six classifiers.

## Data Availability

The methylation datasets analyzed during the current study are available in the NCBI Gene Expression Omnibus (GEO) repository [GSE113501].

## Abbreviations

RCC: Renal cell carcinoma
ccRCC: Clear cell renal cell carcinoma
CpG: Cytosine-phosphate-Guanine
CIMP: CpG island methylator phenotype
PI-CpG: Previously identified CpG
DCA: Directed cluster analysis
HRP: High risk for progress
LRP: Low risk for progress
TNM: Tumor-node metastasis
M0: Non-metastatic (TNM I–III)
M1: Metastatic
TSS: Transcription start site
DM-CpG: Differently methylated CpG
TCGA-KIRC: Tumor Cancer Genome Atlas Kidney Renal Cell Carcinoma
mQTLs: Methylation quantitative trait loci
M0-PF: Non metastatic without tumor progression within five year
M0-P: Non-metastatic tumor with progress within five year
PC: Principal components
CIP: Cumulative incidence of progress
