# Supplementary material for: Combining epigenetic and clinicopathological variables improves specificity in prognostic prediction in clear cell renal cell carcinoma
Source: J Transl Med. 2020 Nov 13;18:435. doi: 10.1186/s12967-020-02608-1 (PMC7666468; doi:10.1186/s12967-020-02608-1)
Supplement: Supplementary file 1 — Additional file 1: Table S1. The Mayo scoring algorithm (6), used in the clinic to estimate the risk of tumor progression. [file 12967_2020_2608_MOESM1_ESM.docx]

**Additional Table S1.** The Mayo **s**coring algorithm (6), used in the clinic to estimate the risk of tumor progression.

|  | Point |
| --- | --- |
| ***T-stage***  T1a  T1b  T2  T3-T4 | 0 p  2 p  3 p  4 p |
| ***Tumor diameter***  ≤ 10 cm  > 10 cm | 0 p  1 p |
| ***N-stage***  NX and N0  N1 | 0 p  2 p |
| ***Fuhrman Grade***  Grade 1-2  Grade 3  Grade 4 | 0 p  1 p  3 p |
| ***Tumor necrosis***  Absent  Present | 0 p  1 p |
|  | |
| ***Risk groups***  Low Risk  Intermediate Risk  High Risk | 0-2 p  3-5 p  ≥ 6 p |
