# Supplementary material for: Combining epigenetic and clinicopathological variables improves specificity in prognostic prediction in clear cell renal cell carcinoma
Source: J Transl Med. 2020 Nov 13;18:435. doi: 10.1186/s12967-020-02608-1 (PMC7666468; doi:10.1186/s12967-020-02608-1)
Supplement: Supplementary file 7 — Additional file 7: Table S7. Classification results for the Mayo scoring classifier (Low Risk or Intermediate/High Risk) and the triple classifier (LRP or HRP) in relation to the true outcome (M0-PF or M0-P). [file 12967_2020_2608_MOESM7_ESM.docx]

**Additional Table S7.** Classification results for the Mayo scoring classifier (Low Risk or Intermediate/High Risk) and the triple classifier (LRP or HRP) in relation to the true outcome (M0-PF or M0-P).

|  |  | **Mayo scoring system** | | | |  |
| --- | --- | --- | --- | --- | --- | --- |
|  |  | **M0-PF,**  **Low Risk** | **M0-PF,**  **Intermediate/High Risk** | **M0-P,**  **Low Risk** | **M0-P,**  **High Risk** | **Total** |
| **Triple classifier** | **M0-PF, LRP** | 25 | 12 | - | - | 37 |
|  | **M0-PF, HRP** | 4 | 17 | - | - | 21 |
|  | **M0-P, LRP** | - | - | 1 | 2 | 3 |
|  | **M0-P, HRP** | - | - | 2 | 15 | 17 |
|  | **Total** | 29 | 29 | 3 | 17 | 78 |
