# Supplementary material for: Combining epigenetic and clinicopathological variables improves specificity in prognostic prediction in clear cell renal cell carcinoma
Source: J Transl Med. 2020 Nov 13;18:435. doi: 10.1186/s12967-020-02608-1 (PMC7666468; doi:10.1186/s12967-020-02608-1)
Supplement: Supplementary file 8 — Additional file 8: Figure S1. Receiver operating characteristic (ROC) analysis and the area under the curve (AUC) analysis based on Mayo scores, and the posterior probabilities of the six classifiers. [file 12967_2020_2608_MOESM8_ESM.pptx]

## Slide 1
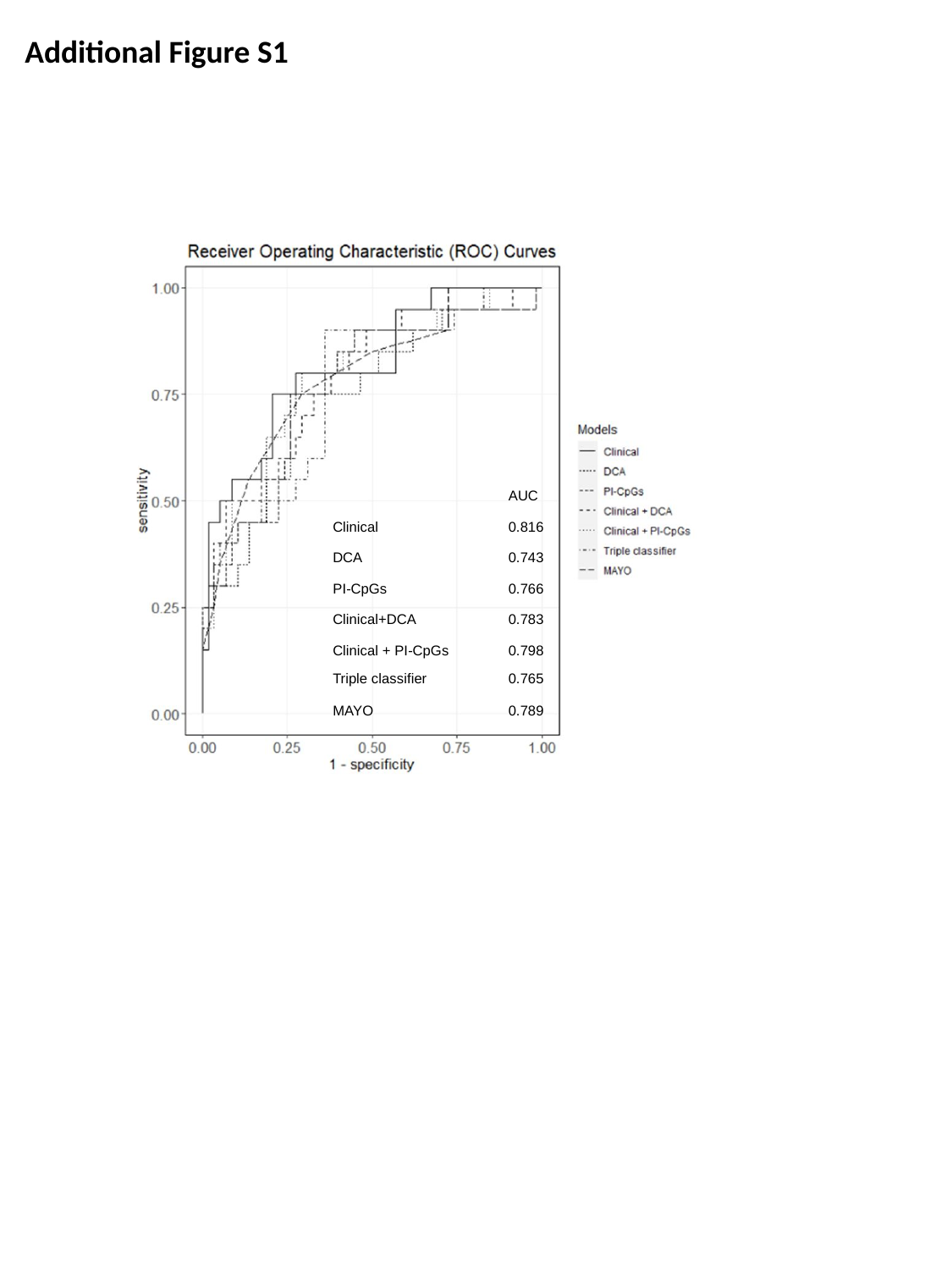

Additional Figure S1
| | AUC |
| --- | --- |
| Clinical | 0.816 |
| DCA | 0.743 |
| PI-CpGs | 0.766 |
| Clinical+DCA | 0.783 |
| Clinical + PI-CpGs | 0.798 |
| Triple classifier | 0.765 |
| MAYO | 0.789 |
